# Experts consensus on the procedure of dental operative microscope in endodontics and operative dentistry

**DOI:** 10.1038/s41368-023-00247-y

**Published:** 2023-09-18

**Authors:** Bin Liu, Xuedong Zhou, Lin Yue, Benxiang Hou, Qing Yu, Bing Fan, Xi Wei, Lihong Qiu, Zhengwei Huang, Wenwei Xia, Zhe Sun, Hanguo Wang, Liuyan Meng, Bin Peng, Chen Zhang, Shuli Deng, Zhaojie Lu, Deqin Yang, Tiezhou Hou, Qianzhou Jiang, Xiaoli Xie, Xuejun Liu, Jiyao Li, Zuhua Wang, Haipeng Lyu, Ming Xue, Jiuyu Ge, Yi Du, Jin Zhao, Jingping Liang

**Affiliations:** 1 Department of Endodontics and Operative Dentistry, Shanghai Ninth People’s Hospital, Shanghai Jiao Tong University School of Medicine; College of Stomatology, Shanghai Jiao Tong University; National Center for Stomatology; National Clinical Research Center for Oral Diseases; Shanghai Key Laboratory of Stomatology, Shanghai Research Institute of Stomatology, Shanghai, China; 2 State Key Laboratory of Oral Diseases & National Center for Stomatology & National Clinical Research Center for Oral Diseases & Department of Operative Dentistry and Endodontics, West China Hospital of Stomatology, Sichuan University, Chengdu, China; 3 Department of Cariology and Endodontology, Peking University School and Hospital of Stomatology & National Center of Stomatology & National Clinical Research Center for Oral Diseases & National Engineering Laboratory for Digital and Material Technology of Stomatology & Beijing Key Laboratory of Digital Stomatology & Research Center of Engineering and Technology for Computerized Dentistry Ministry of Health & NMPA Key Laboratory for Dental Materials, Beijing, China; 4 Department of Endodontics and Operative Dentistry, Beijing Stomatological Hospital, Capital Medical University, Beijing, China; 5 Department of Operative Dentistry and Endodontics, School of Stomatology, The Fourth Military Medical University, Xi’an, China; 6 State Key Laboratory of Oral & Maxillofacial Reconstruction and Regeneration, Key Laboratory of Oral Biomedicine Ministry of Education, Hubei Key Laboratory of Stomatology, School & Hospital of Stomatology, Wuhan University, Wuhan, China; 7 Department of Operative Dentistry and Endodontics, Hospital of Stomatology, Guanghua School of Stomatology, Sun Yat-Sen University & Guangdong Provincial Key Laboratory of Stomatology, Guangzhou, China; 8 Department of Endodontics, School of Stomatology, China Medical University, Shenyang, China; 9 Stomatology Hospital, School of Stomatology, Zhejiang University School of Medicine, Zhejiang Provincial Clinical Research Center for Oral Diseases, Key Laboratory of Oral Biomedical Research of Zhejiang Province, Cancer Center of Zhejiang University, Hangzhou, China; 10 School and Hospital of Stomatology, Fujian Medical University, Fuzhou, China; 11 Department of Endodontics, Stomatological Hospital of Chongqing Medical University, Chongqing, China; 12 Department of Cariology and Endodontics, College of Stomatology, Xi’an Jiaotong University, Xi’an, China; 13 Department of Endodontics, Affiliated Stomatology Hospital of Guangzhou Medical University, Guangdong Engineering Research Center of Oral Restoration and Reconstruction, Guangzhou Key Laboratory of Basic and Applied Research of Oral Regenerative Medicine, Guangzhou, China; 14 Department of Cariology and Endodontics, Xiangya Stomatological Hospital, Central South University, Changsha, China; 15 Special Clinic Department of Stomatology, The First Affiliated Hospital of Zhengzhou University, Zhengzhou, China; 16 Nanjing Stomatological Hospital, Affiliated Hospital of Medical School, Nanjing University, Nanjing, China; 17 Jinan Stomatological hospital, Jinan, China; 18 The First Affiliated Hospital of Xinjiang Medical University (Affiliated Stomatology Hospital), Urumqi, China

**Keywords:** Dental treatments, Endodontics, Restorative dentistry

## Abstract

The dental operative microscope has been widely employed in the field of dentistry, particularly in endodontics and operative dentistry, resulting in significant advancements in the effectiveness of root canal therapy, endodontic surgery, and dental restoration. However, the improper use of this microscope continues to be common in clinical settings, primarily due to operators’ insufficient understanding and proficiency in both the features and established operating procedures of this equipment. In October 2019, Professor Jingping Liang, Vice Chairman of the Society of Cariology and Endodontology, Chinese Stomatological Association, organized a consensus meeting with Chinese experts in endodontics and operative dentistry. The objective of this meeting was to establish a standard operation procedure for the dental operative microscope. Subsequently, a consensus was reached and officially issued. Over the span of about four years, the content of this consensus has been further developed and improved through practical experience.

## Introduction

The human eye possesses a versatile optical mechanism capable of accommodating various visual requirements. By means of contracting or relaxing the ciliary muscles, the convexity of the lens is altered, enabling the formation of clear images of objects situated at different distances on the retina. As objects decrease in size and increase in distance from the eye, the angle at which they are projected onto the retina diminishes. Consequently, finer details can solely be discerned when objects are in closer proximity to the eye, resulting in a larger angle of projection onto the retina. One effective approach involves positioning the visual object in close proximity to the eye, accompanied by the contraction of the ciliary muscles to enhance the convexity of the lens, thereby facilitating the perception of intricate details. Consequently, dentists frequently find themselves bending over the patient’s oral cavity to address the minuscule teeth, as the human eye possesses restricted adaptability and experiences a decline in its capacity to accommodate close-range objects with advancing age. Nonetheless, the utilization of the Dental Operative Microscope (DOM) presents a viable solution for dentists to surmount this constraint. Firstly, the utilization of the DOM enhances the discernment of intricate structures, thereby facilitating improved discrimination. Secondly, the DOM affords the opportunity for ocular relaxation and mitigates the occurrence of ciliary muscle fatigue, necessitating minimal adaptation. Thirdly, the DOM promotes spinal relaxation for dentists, enabling a more ergonomic and comfortable seated posture.

The year 1981 marked the introduction of the first commercially available DOM known as “Dentiscope”.^1,2^ Over the course of four decades, DOM has gained widespread utilization within the field of dentistry. While endodontics remains the primary discipline employing DOM, other specialties such as periodontics, implantology, and prosthodontics are swiftly incorporating microscopic magnification techniques.^3–8^ The American Association of Endodontists (AAE) played an early and influential role in promoting microscopy training for endodontic residents. Their efforts resulted in the successful inclusion of a microscope proficiency standard within the educational requirements for postgraduate endodontic programs by the Commission on Dental Accreditation (CODA) in 1998.^2,9^ In 2016, the Society of Cariology and Endodontology, Chinese Stomatological Association established and released guidelines for the utilization of microscopes in endodontics, which have significantly influenced the standardized implementation of DOM in endodontic treatment within China.^10^ Nonetheless, the absence of comprehensive instructions and application specifications has resulted in frequent misunderstandings and improper utilization of DOM among dental practitioners, thereby diminishing its potential value in clinical practice. In October 2019, an expert seminar was organized by Professor Jingping Liang, Vice Chairman of the Society of Cariology and Endodontology, Chinese Stomatological Association, with the aim of refining and standardizing the application of DOM in the diagnosis and treatment of endodontics and operative dentistry. Subsequently, a set of consensus statements were reached and published.^11^ Over the course of about four years, through practical experience, the content of the consensus has been enhanced and can now be summarized as follows.

## Basic instruments required for microscopic endodontics and operative dentistry

DOMs offer enhanced visualization capabilities, presenting amplified, brightened, and more distinct images that enable a higher level of detail for improved observation. Consequently, this facilitates refined dental procedures, minimizing potential harm to the tooth structure during preparation and treatment. However, the successful execution of precise operations with DOMs relies on the aid and backing of diverse purpose-built instruments, ensuring accurate diagnosis and treatment. It is worth mentioning that the instruments utilized for treatment under a microscope exhibit a progressive enhancement in their design, a heightened specialization in their functions, and a diversification in their types. Certain devices have incorporated a matte or black surface treatment to minimize light reflection, thereby enhancing the safety and comfort of the practitioner during treatment. Furthermore, the utilization of conventional instrumentation is also necessary to support microscopic treatment, with the specific devices required varying depending on the nature of the treatment being performed.^10,12–16^

### Microscopic endodontics

Rubber dam isolation system, front-surface dental mirror, endodontic explorer DG-16, micro-opener, nickel-titanium rotary instruments, ultrasonic units and tips (including root canal irrigation tips, root canal access preparation tips and non-surgical endodontic re-treatment tips), warm gutta-percha vertical condensation system, etc.

### Endodontic microsurgery

Examination/inspection instruments (e.g., front-surface dental mirror, micromirror, apical explorer MEX1, periodontal probe), incision and elevation instruments (e.g., microsurgical blade or 15 C surgical blade and handle, soft tissue periosteal elevators), tissue retraction instruments, osteotomy instruments (e.g., 45° surgical handpiece with a Lindemann bur), curettage instruments (periodontal curettes, surgical curettes, and mini-endodontic curettes), ultrasonic units and tips for root-end preparation, micro irrigator/drier (e.g., stropko irrigator), microplugger and burnisher instruments, micro suturing instruments (e.g., castroviejo needle holder, micro scissors), etc.

### Operative dentistry under a microscope

Rubber dam isolation system, minimally invasive burs and instruments, instruments dedicated to composite resin placement, condensation, adaptation, and contouring, instruments for finishing and polishing of composite resin restorations, etc.

## Scope of the application of DOM

Endodontics, as well as operative dentistry, are the main disciplines in which DOMs are used in dentistry. The application of DOM in endodontics and operative dentistry mainly focuses on diagnosis and treatment.^10,12–14,17,18^

### Application scope of DOM in diagnosis

DOM mainly plays a supporting role in diagnosis, including the auxiliary diagnosis of caries, such as the early diagnosis of superficial caries, the conformation of marginal secondary caries, the detection of approximal caries, and the determination of pulp exposure in deep caries; the auxiliary diagnosis of non-carious diseases, such as the identification of cracked teeth, the exploration and confirmation of root fracture in apical surgery, etc.

### Application scope of DOM in treatment

DOM has a broader range of therapeutic application scope in endodontics and operative dentistry, including but not limited to dental restoration and microscopic endodontics such as root canal treatment, non-surgical endodontic re-treatment, and endodontic microsurgery. The recommended use of DOM in treatment mainly includes:

1. Root canal treatment: (1) access preparation (removal of the existing restorations); (2) identification of the inner anatomical structure of the pulp chamber; (3) location of sclerosed canals; (4) identification and assessment of variant root canals; (5) observation during root canal irrigation; (6) assurance of optimal obturation; (7) cleaning the residual sealer in the pulp chamber after root canal obturation; (8) the delivery and placement of bioceramic materials in apical barrier technique, etc.
2. Non-surgical endodontic re-treatment: (1) access preparation (removal of the existing restorations, root posts, core materials, etc.); (2) location of missed canals; (3) removal of existing root filling materials; (4) removal of separated instruments; (5) observation during and after root canal re-preparation; (6) assurance of optimal re-obturation, etc.
3. Endodontic microsurgery: (1) management of soft and hard tissues in apical surgery: incision, flap elevation, osteotomy, periapical curettage, root-end resection, root-end preparation, root-end filling, flap reposition, suturing, etc.; (2) evaluation and repair of perforations, etc.
4. Dental restoration: (1) minimally invasive caries removal and cavity preparation; (2) accurate replacement of the existing restorations; (3) restoration with composite resin (matrix adaptation, layering and filling, marginal adaption, finishing and polishing); (4) tooth preparation for indirect restorations such as inlays, onlays, full crown restorations, etc.

DOM can assist in diagnosis and treatment and improve outcomes.^19–24^ However, it should be stressed that the role of DOM should not be artificially exaggerated, and its indications should not be expanded indefinitely. It is not always necessary to use DOM for the entire treatment process.

### Other application scope of DOM

Equipped with a digital image, video acquisition system, and wireless transmission system, DOM can photograph and film the treatment process, enabling (1) simultaneous collection and preservation of clinical data; (2) real-time wireless transmission of video and images; (3) on-site or distance education of microscopic operation skills; (4) remote consultation of difficult cases; and (5) convenient communication and exchange between doctors and patients.

## Application of DOM in endodontics and operative dentistry

Basic requirements

In order to promote the uniform utilization of DOM and fully exploit its benefits, the implementation of four-handed dentistry and rubber dam isolation is recommended.

Prior to employing DOM, dentists are required to meet certain prerequisites.

- Have an intimate knowledge of the structure and function of DOM.
- Know the pupil distance of himself and the diopter adjustment well.
- Be familiar with the correct use of DOM.
- Master the rubber dam isolation technique.
- Master four-handed dentistry.
- Be acquainted with the diagnosis skills and treatment techniques of related diseases.
- Complete pre-operatively the necessary evaluation of the patient’s whole body, mouth, temporomandibular joint, teeth, etc.

Additionally, while utilizing DOM, dentists are expected to fulfill specific demands.

- Sit-in posture conforms to ergonomics.
- Ensure hand-eye coordination under a microscope.
- Choose the appropriate magnification.
- Be acquainted with the use of instruments under DOM.

Furthermore, it is essential for assistants to acquire proficiency in four-handed dentistry within the context of DOM. It is worth noting that precautions must be taken to prevent eye injuries resulting from the excessively bright light emitted by DOM during its use, necessitating the use of goggles by both assistants and patients. Lastly, it is advisable to limit the duration of DOM usage to avoid potential adverse effects.^10,12,13^

### Position of dentist

**Fig. 1:**
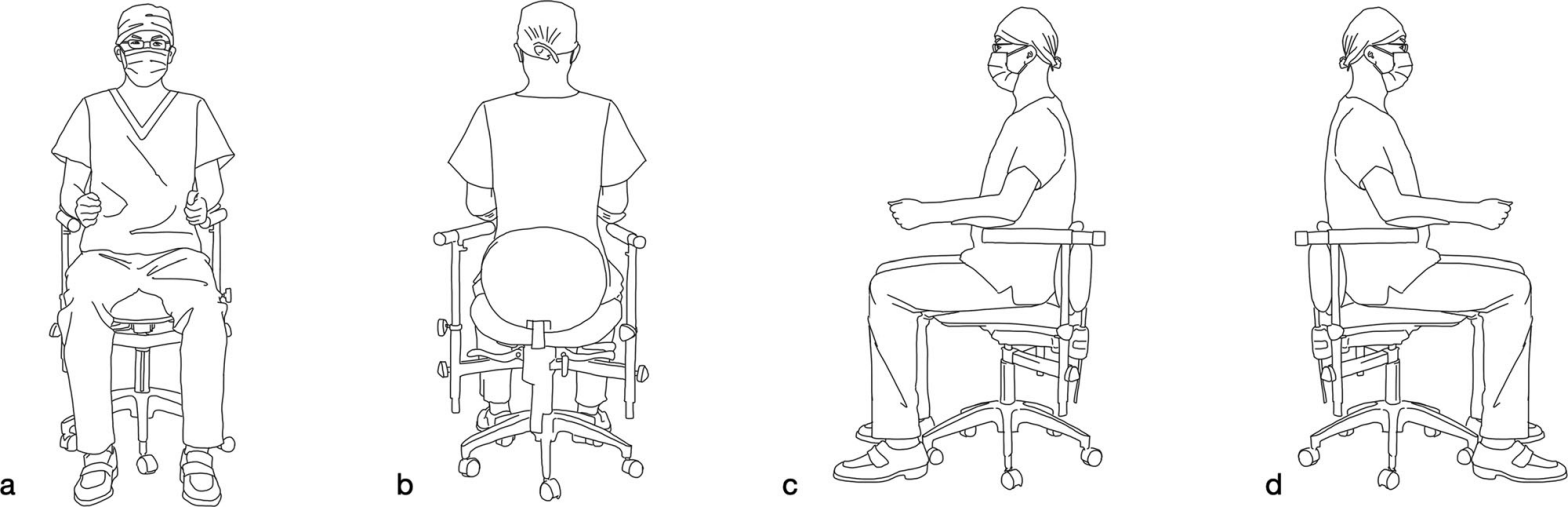
Demonstration of the basic postural requirements for the dentist (Citing and modifying from Liu, B. & Liang, J. P. *Atlas of the Operation of Dental Operative Microscope in Endodontics and Operative Dentistry*. 1st edn, Shanghai Jiao Tong University Press, 2020). **a** View from the dentist’s front. **b** View from the dentist’s back. **c** View from the dentist’s left side. **d** View from the dentist’s right side

During the execution of treatment under a microscope, the maintenance of a proper seated position can significantly alleviate the strain on the practitioner’s cervical vertebrae and spinal column during extended periods of sitting. The postural demands are essentially identical for microscopic endodontic treatment and operative dentistry under a microscope, while endodontic microsurgery necessitates a more superior operative field of view and visualization angle, resulting in slightly distinct postural requirements for the dentist. This discrepancy primarily manifests in the relative positioning of the dentist in relation to the patient. The general guidelines for the seated posture of the dentist are outlined as follows.^12,13,16,17,25^ (Fig. 1).

- Head keep upright.
- Eyes flat eyepieces.
- Spine vertical on the ground.
- Upper arms naturally down.
- Forearms flat on the elbow rests.
- Elbows close to the torso.
- Hands equal to the operative area.
- Thighs parallel to the ground.
- Calves vertical on the ground.
- Feet flat on the ground.

**Fig. 2:**
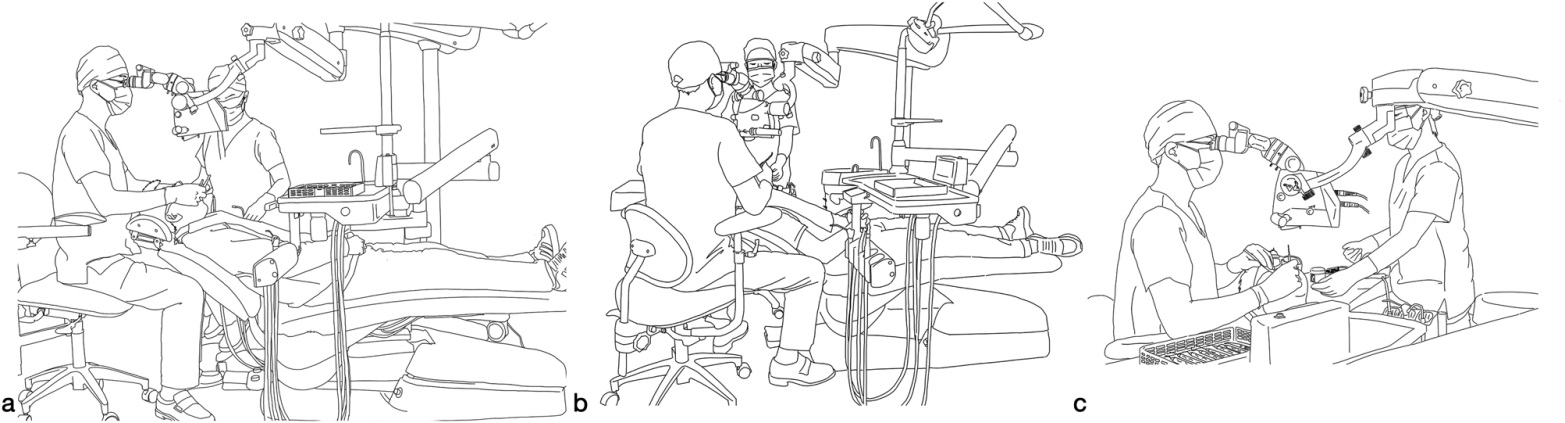
Demonstration of the proposed positions of the dentist, assistant, and patient in microscopic endodontic treatment (Citing and modifying from Liu, B. & Liang, J. P. *Atlas of the Operation of Dental Operative Microscope in Endodontics and Operative Dentistry*. 1st edn, Shanghai Jiao Tong University Press, 2020). **a** Microscopic endodontic treatment in lower anterior teeth (view from the dentist’s right side). **b** Microscopic endodontic treatment in lower right posterior teeth (view from the dentist’s back). **c** Microscopic endodontic treatment in lower right posterior teeth (view from the dentist’s right side)

**Fig. 3:**
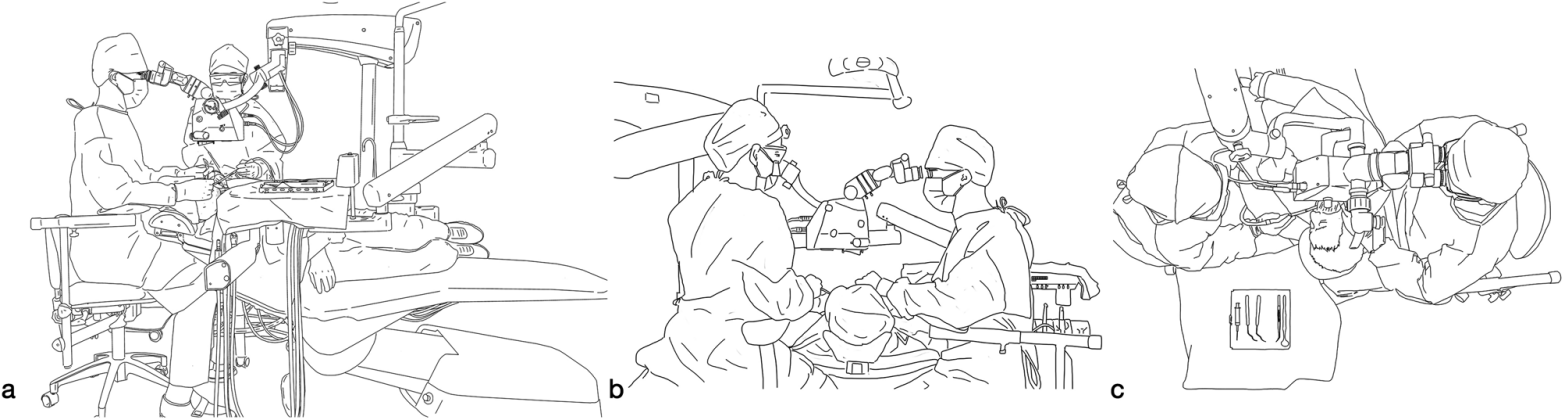
Demonstration of the proposed positions of the dentist, assistant, and patient in endodontic microsurgery (Citing and modifying from Liu, B. & Liang, J. P. *Atlas of the Operation of Dental Operative Microscope in Endodontics and Operative Dentistry*. 1st edn, Shanghai Jiao Tong University Press, 2020). **a** Endodontic microsurgery in the lower left posterior teeth (view from the dentist’s right side). **b** Endodontic microsurgery in lower right posterior teeth (view from the dentist’s left side). **c** Endodontic microsurgery in lower right posterior teeth (view from above the dentist’s head)

In dental practice, it is customary for the dentist to typically occupy the 12 o’clock position, situated behind the patient’s head, or alternatively, the 9 o’clock position on the right side. The dentist retains the flexibility to adjust their position within the approximate range of 9 to 12 o’clock as required, and during apical surgery, they have a relatively broader range of movement within the treatment area.^4,12,16,25^ (Figs. 2 and 3).

### Position of patient

**Fig. 4:**
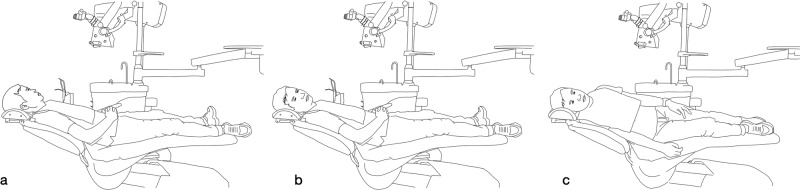
Demonstration of the basic postural requirements for the patient (Citing and modifying from Liu, B. & Liang, J. P. *Atlas of the Operation of Dental Operative Microscope in Endodontics and Operative Dentistry*. 1st edn, Shanghai Jiao Tong University Press, 2020). **a** patient lying in a flat position. **b** Patient in the flat position with his head tilted to the right side. **c** Patient lying on the right side

Once the dentist has assumed the appropriate seating position, it is imperative to establish a suitable treatment position for the patient. Typically, patients assume a comfortable, semi-supine, or supine posture. Given the necessity of an optimal visual field during procedures conducted under a microscope, contingent upon the angle of the operative region, adjustments to the patient’s head position are required to ensure that the dental mirror aligns at an approximate 45° angle with the microscope’s coaxial light. This alignment facilitates the production of a clear, reflected image with the aid of the dental mirror. Depending on the positioning of the teeth, it may be necessary to tilt the patient’s head to one side. In cases involving apical microsurgery of posterior teeth, the patient may be positioned in a lateral lying position as deemed necessary^12,14^ (Fig. 4).

### Position of assistant

In four-handed dentistry, the primary responsibility of the dental assistant is to facilitate the delivery of instruments and materials, as well as the removal of fluid from the patient’s oral cavity. The fundamental objective is to ensure that the dentist maintains an unobstructed view of and convenient access to the treatment area without any hindrance to his or her work. Consequently, it is generally recommended for the assistant to be positioned on the left side of the patient, within the 2–4 o’clock range, facing the dentist, and maintaining an approximate viewing height of 10 cm above the dentist. The proper positioning for the assistant during treatment involves placing their feet on the footrest of the chair, ensuring that their hips are level with the patient’s shoulders and their thighs are parallel to the floor. Additionally, the line of the assistant’s shoulders should be parallel to the line formed by the patient’s left ear and left shoulder at an approximate angle of 45° to the long axis of the patient’s body. Throughout the treatment, the assistants should strive to position themselves as close as possible to the delivery area, maintaining a parallel alignment with the practitioner and facing slightly to the left. The positioning of the assistant may require adjustment based on the treatment modality and the alignment of the teeth^12,14^ (Figs. 2 and 3).

### Position of DOM

After establishing the fundamental positions of the dentist, patient, and assistant, it becomes imperative to appropriately position the DOM, set the accurate working distance, and perform the initial focusing. The working distance of the DOM denotes the space between the objective lens’s surface and the object within the treatment field, wherein the object is perceived with utmost clarity under the microscope. The working distance is contingent upon the focal length of the objective lens, whereby a longer focal length corresponds to a greater working distance. The fixed-focus objective lens possesses a constant focal length, typically indicated on the lens mount, such as *f* = 200 mm, which closely corresponds to the working distance of the lens. In order to attain a sharp image, it is necessary for the objective lens to maintain a working distance of approximately 200 mm from the object, placing the object within the lens’s focal point. In practical application, adjusting the magnification to its lowest setting and manipulating the depth of focus mechanism allows for the attainment of the appropriate working distance in accordance with the focal length of the objective lens, thereby facilitating initial focusing on the object. The working distances commonly employed in DOM are 200, 250, and 300 mm. In contrast, a zoom lens possesses a variable focal length range (e.g., 200–350 mm) that can be tailored to diverse applications and ergonomic demands by selecting appropriate working distances. The utilization of a zoom lens facilitates effortless adjustment of the microscope’s working distance, enabling precise focus attainment through manipulation of the focus adjustment knob rather than physically raising or lowering the microscope. The maintenance of proper posture during DOM employment is intricately linked to the judicious selection of the working distance. In the event that the working distance is insufficient, the practitioner may be compelled to assume an erroneous posture during the utilization of DOM, characterized by an arched back, lowered head, and forward tilting of the upper body. Conversely, if the working distance is excessive, the practitioner may be compelled to assume an erroneous posture, characterized by an elevated head and backward tilting of the upper body. Inadequate working distances have the potential to strain the practitioner’s back and/or neck muscles, resulting in discomfort or even injury following prolonged usage of the microscope.

### Select the appropriate magnification

The magnification of a DOM is primarily influenced by the magnification of the ocular lens and the objective lens. However, the final magnification is not simply the product of these two lens magnifications. The calculation for determining the overall magnification of the DOM is expressed as *M*_*T*_ = (*f*_*t*_/*f*_*o*_) *M*_*e*_ *M*_*c*_, where *M*_*T*_ denotes the total magnification of the DOM, *f*_*t*_ represents the focal length of the ocular lens, *f*_*o*_ represents the focal length of the objective lens, *M*_*e*_ represents the magnification of the ocular lens, and *M*_*c*_ represents the magnification factor displayed on the magnification changer. Hence, it is imperative to concurrently measure the focal lengths of both the ocular lens and the objective lens while documenting the magnification in clinical procedures. The calculation formula is solely applicable to DOMs possessing a constant focal length, rendering it unsuitable for DOMs with a variable focal length due to the disparity between the working distance and the focal distance.^12,25^

The magnification of DOM typically ranges from 3 to 30 times, with certain models capable of achieving even higher magnification levels. In general, magnifications ranging from 3 to 8 are classified as low magnification, characterized by high light intensity, enabling a wide field of view and a substantial depth of field. This range is suitable for observing the entire tooth, cavity, and operative area. Magnifications between 9 and 16 fall under the category of medium magnification, offering a moderate visual field range, depth of field, and brightness, making it suitable for most clinical procedures. Magnifications exceeding 16 are considered high magnification, resulting in a narrower field of view and a reduced depth of field. Due to the significantly limited effective aperture of the DOM, there is a substantial reduction in the quantity of incoming light, thereby necessitating high lighting conditions. Consequently, the DOM proves to be well-suited for the observation of intricate structures pertaining to affected teeth, root canals, root surfaces, and apical regions.^12,15,25^

### Use specific filters during the operation

To accommodate diverse application scenarios and environments, the light source of DOM is furnished with specialized filters, the yellow filter and the green filter. Dentists have the discretion to opt for the use of filters during treatment based on treatment requirements.

The yellow filter is primarily employed in the light-curing composite resin restoration process to prevent premature curing of the resin surface due to the intense light emitted by DOM, thereby avoiding any negative impact on the therapeutic outcome during treatment. Differently, the green filter serves the purpose of diminishing the presence of red hues within the entire visual field, thereby alleviating visual fatigue and augmenting the distinction between blood-filled and bloodless tissues. This enhancement, in contrast, facilitates the observation of intricate tissue and organ structures, rendering it particularly suitable for endodontic surgery and other surgical interventions.

### Recommendations for the general steps of using DOM

The standardized use of DOM should be carried out in accordance with certain operating procedures.^12,13,26^ The recommended general steps for using DOM are as follows.

- Isolate and protect the knobs and handles, as well as any other parts of the DOM that are susceptible to contamination before use.
- The dentist and assistant assume an adequate seated position.
- Ensure that the dental chair and patient are positioned correctly.
- Position DOM properly.
- Adjust the eyepiece distance according to the dentist’s interpupillary distance.
- Set the diopter correction according to the dentist’s eye correction value.
- Fine-adjust the patient’s head position to ensure the operation area is centered in the visual field.
- Set the appropriate magnification and fine focusing.
- Adjust the magnification, brightness, and pinhole aperture, and use specific filters during operation for different purposes.

## Conclusions and expectations

It has been more than four decades since the introduction of the DOM, which was initially utilized in endodontics and has since expanded its application to encompass nearly all branches of dentistry. The DOM offers enhanced visualization through magnified images, a brighter field of view, and precise maneuverability. Consequently, it has not only revolutionized the diagnostic and therapeutic approaches employed by dental practitioners but has also transformed patients’ perceptions of dental treatment while simultaneously elevating the standard of clinical diagnosis and treatment. As microscopy and microscope equipment continue to advance, it is imperative for dentists and their assistants who utilize DOM to possess a comprehensive understanding of the microscope’s structure and functionality. Additionally, they should acquire proficiency in operating the microscope and adhere to standardized techniques in order to effectively harness the benefits of DOM and maximize its potential as a formidable clinical tool.
